# Estimation of inspiratory effort using airway occlusion maneuvers in ventilated children: a secondary analysis of an ongoing randomized trial testing a lung and diaphragm protective ventilation strategy

**DOI:** 10.1186/s13054-023-04754-6

**Published:** 2023-11-29

**Authors:** Yukie Ito, Matías G. Herrera, Justin C. Hotz, Miyako Kyogoku, Christopher J. L. Newth, Anoopindar K. Bhalla, Muneyuki Takeuchi, Robinder G. Khemani

**Affiliations:** 1 Department of Intensive Care, Osaka Women’s and Children’s Hospital, Osaka, Japan; 2 Department of Anesthesiology and Critical Care Medicine, Children’s Hospital Los Angeles, Los Angeles, USA; 3 Department of Intensive Care, Hospital de Pediatría JP Garrahan, Buenos Aires, Argentina; 4 Department of Pediatrics, University of Southern California Keck School of Medicine, Los Angeles, USA

**Keywords:** Mechanical ventilation, Artificial respiration, Work of breathing, Ventilator-induced lung injury, Myotrauma

## Abstract

**Background:**

Monitoring respiratory effort in ventilated patients is important to balance lung and diaphragm protection. Esophageal manometry remains the gold standard for monitoring respiratory effort but is invasive and requires expertise for its measurement and interpretation. Airway pressures during occlusion maneuvers may provide an alternative, although pediatric data are limited. We sought to determine the correlation between change in esophageal pressure during tidal breathing (∆Pes) and airway pressure measured during three airway occlusion maneuvers: (1) expiratory occlusion pressure (Pocc), (2) airway occlusion pressure (P0.1), and (3) respiratory muscle pressure index (PMI) in children. We also sought to explore pediatric threshold values for these pressures to detect excessive or insufficient respiratory effort.

**Methods:**

Secondary analysis of physiologic data from children between 1 month and 18 years of age with acute respiratory distress syndrome enrolled in an ongoing randomized clinical trial testing a lung and diaphragm protective ventilation strategy (REDvent, R01HL124666). ∆Pes, Pocc, P0.1, and PMI were measured. Repeated measure correlations were used to investigate correlation coefficients between ∆Pes and the three measures, and linear regression equations were generated to identify potential therapeutic thresholds.

**Results:**

There were 653 inspiratory and 713 expiratory holds from 97 patients. Pocc had the strongest correlation with ∆Pes (*r* = 0.68), followed by PMI (*r* = 0.60) and P0.1 (*r* = 0.42). ∆Pes could be reliably estimated using the regression equation ∆Pes = 0.66 $\times$ Pocc (*R*^2^ = 0.82), with Pocc cut-points having high specificity and moderate sensitivity to detect respective ∆Pes thresholds for high and low respiratory effort. There were minimal differences in the relationship between Pocc and ∆Pes based on age (infant, child, adolescent) or mode of ventilation (SIMV versus Pressure Support), although these differences were more apparent with P0.1 and PMI.

**Conclusions:**

Airway occlusion maneuvers may be appropriate alternatives to esophageal pressure measurement to estimate the inspiratory effort in children, and Pocc represents the most promising target.

*Trial registration*: NCT03266016; August 23, 2017.

**Supplementary Information:**

The online version contains supplementary material available at 10.1186/s13054-023-04754-6.

## Introduction

Controlled mechanical ventilation with minimal patient effort has been a strategy to minimize the risk for ventilator-induced lung injury in patients with acute respiratory distress syndrome (ARDS) [1–4]. It is increasingly recognized that this strategy may cause harm if respiratory effort is excessively low, leading to ventilator-induced diaphragm dysfunction (VIDD) [5, 6]. On the other hand, if respiratory effort is excessively high, it can exacerbate lung stress and strain, and cause patient self-inflicted lung injury (P-SILI) [7, 8]. Maintaining physiologic levels of patient effort of breathing during mechanical ventilation is a therapeutic target under investigation, to balance lung and diaphragm protective ventilation simultaneously [9].

The gold standard method to estimate patient effort of breathing is esophageal manometry, which estimates pleural pressure, but has several limitations. Esophageal manometry is invasive, requires a special catheter, and requires expertise for appropriate catheter placement, calibration, and interpretation [10, 11]. A less invasive alternative to esophageal manometry may include measures of airway pressure during airway occlusion, which can be performed on nearly any ventilator. Expiratory occlusion pressure (Pocc) and airway occlusion pressure (P0.1) are measured with inspiratory effort during an end-expiratory occlusion on the ventilator [12–14], while respiratory muscle pressure index (PMI) is measured during an end-inspiratory occlusion, when the patient relaxes [15–17]. While these parameters are related, they each represent different physiologic concepts. Peak-to trough esophageal pressure during tidal breathing (∆Pes) and Pocc reflect total patient effort, both resistive and elastic work [4, 12, 18]. PMI reflects the patient’s contribution to the elastic work in the respiratory system, needed to move the tidal volume [15, 18]. P0.1 represents a combination of respiratory drive and respiratory effort [13, 14]. There have been some studies in adult patients identifying relatively strong correlations between these parameters and esophageal manometry derived measures of effort of breathing [12, 14, 15, 19, 20], although comparative data amongst the maneuvers, and pediatric data are minimal [21]. The optimal cut points to identify low or excessive respiratory effort with each of these parameters also remains an important question, although some targets have been suggested [9].

There are almost no data about the accuracy of any of these maneuvers in children, which is particularly important given substantial differences across the pediatric age spectrum with respect to chest wall elastance, airway resistance, and control of respiratory drive..

## Materials and methods

The primary objective of this study was to characterize the correlation between ∆Pes and each of the following: Pocc, P0.1, PMI in mechanically ventilated children. Secondary objectives focused on finding the respective thresholds for Pocc, P0.1, and PMI that detect excessively high and low inspiratory effort.

We performed secondary analysis of physiologic data from children on pressure control (PC) or pressure support (PS) ventilation, enrolled in an ongoing randomized trial testing a lung and diaphragm protective ventilation strategy (REDvent, R01HL124666) (Clinical Trials. gov NCT03266016) that uses esophageal manometry at Children’s Hospital Los Angeles (CHLA) [22]. The protocol has been approved by the CHLA Institutional Review Board as well as an independent Data Safety and Monitoring Board. All patients were enrolled in the parent REDvent study and were between 1 month and 18 years of age, and met hypoxemia criteria for pediatric ARDS [23]. In all patients, informed consent was obtained. Detailed inclusion and exclusion criteria are provided in the supplement.

**Fig. 1:**
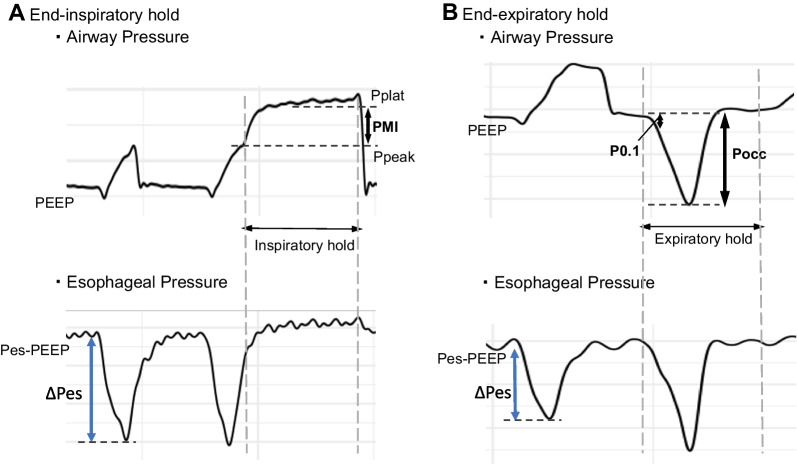
Physiologic waveforms of airway pressure and esophageal pressure during the end-inspiratory (**A**) and end-expiratory hold (**B**). The elements of airway and esophageal pressure used for analysis are as follows. Peak pressure (Ppeak): the highest airway pressure before the start of an end-inspiratory hold. Plateau pressure (Pplat): the airway pressure that reached a plateau during an end-inspiratory hold. Respiratory muscle pressure index (PMI): Pplat minus Ppeak. Airway occlusion pressure (P0.1): the drop in airway pressure during expiratory occlusion from the beginning of the drop to 100 ms after the first drop in airway pressure. Expiratory occlusion pressure (Pocc): the difference from positive end-expiratory pressure (PEEP) to the lowest airway pressure during the first inspiratory cycle during an expiratory hold maneuver. Delta Pes (∆Pes): the difference from end-expiratory Pes to maximum negative Pes during non-occluded (PC or PS) breaths. **A** In airway occlusion at the end of inspiration, if the patient became relaxed during occlusion, Pplat is achieved. The difference between the plateau pressure and Ppeak is the PMI. **B** In airway occlusion at the end of expiration, the maximum negative pressure during the next spontaneous breath is Pocc, and the negative pressure 0.1 ms after the start of inspiration is P0.1

Physiologic waveforms of flow, esophageal pressure (Pes) and airway pressure (Paw) were recorded once daily. All patients were intubated with a cuffed endotracheal tube. For each day the patient remained intubated, a target of 3 inspiratory and 3 expiratory hold maneuvers were performed by using occlusion buttons on the ventilators. For analysis, all patients had to have evidence of spontaneous breathing, measured by negative deflection of the esophageal pressure waveform during inspiration. We used median values of the measures obtained from up to 3 inspiratory and expiratory occlusion maneuvers and the median ΔPes in up to 3 spontaneous PC and/or PS breaths at a time point near the occlusion maneuvers per test day. Data were selected for analysis at the waveform level, and inappropriate waveforms were excluded (Additional file 2). Ventilators included Servo I (Maquet, Solna, Sweden), NKV-550 (OrangeMed, Santa Ana, CA), or AVEA (CareFusion, Yorba Linda, CA) ventilator. All test days using the AVEA were later excluded because it was found that the AVEA ventilator does not allow airway pressure to rise above a set peak inspiratory pressure during an inspiratory hold, which invalidates PMI measurements. The elements of airway and esophageal pressure used for the analysis are described in Fig. 1.

### Protocol for monitoring

Airway pressure (Paw) was measured with a proximal sampling line placed just after the endotracheal tube, along with a self-calibrating pneumotachometer (Viasys Variflex 51,000–40094; Conshohocken, PA). One of the three esophageal catheters were used, based on the size of the patient (Carefusion, Avea SmartCath 6, 7, or 8 Fr). The amount of air inflated into esophageal balloon was determined before each measurement using a previously validated calibration algorithm [1] All sensors were connected to a custom-made hardware device (New Life Box, Applied Biosignals, Weener, Germany), which recorded data at a frequency of 200 Hz. The data were then post-processed with a custom-built software program for breath annotation using R (R Core Team, Vienna, Austria). All measurements of P0.1, Pocc or PMI were computed in the post-processing software.

### Analysis

For analyses evaluating data which were independent between groups, Kruskall-Wallis or Pearson's chi-square tests were performed with Bonferroni correction for multiple comparisons. For data that was not independent (i.e., measurements on multiple days from the same patient), we chose one observation per patient at random or used linear mixed models to first control for patient level effects. Similarly, repeated measures correlation was used with log or cubed transformation as necessary to satisfy assumptions of normality. Dose response relationships between ∆Pes and suggested cut-offs of Pocc, P0.1, and PMI are reported with box-plots [9, 21]. Subgroup analyses were performed based on mode of ventilation (Synchronized Intermittent Mandatory Ventilation Pressure Control with Pressure Support (SIMV PC-PS) or Pressure Support Ventilation (PSV)) and age (< 1 year, 1 year to < 9 years, >  = 9 years to <  = 18 years). Additional sensitivity analyses were performed based on adequacy of calibration with the esophageal catheter, using respiratory muscle pressure (Pmusc) instead of ∆Pes, and evaluating the relationship between patient effort on PC versus PS breaths when on SIMV (Additional file 2).

Several thresholds have been proposed using esophageal pressure to characterize high or low inspiratory effort [9]. For analysis, we evaluated three potential thresholds to characterize high effort (∆Pes > 12, 15, 20 cmH_2_O) and 2 thresholds to characterize low inspiratory effort (∆Pes < 3, 5 cmH_2_O). We developed regression equations for the relationship between ∆Pes and Pocc, P0.1, and PMI, respectively, and report the respective values for each parameter which correspond to the proposed high and low effort ∆Pes thresholds. We then describe the sensitivity and specificity of each of the values to detect high and low effort [9].

In addition, the following data were collected on all test days: respiratory mechanics (resistance, peak flow, tidal volume, static respiratory system compliance (C_RS_), chest wall compliance (C_CW_), lung elastance (E_L_)/ respiratory system elastance (E_RS_)); respiratory drive (pH, pCO_2_, respiratory rate (actual RR), State Behavioral Scale (SBS) [24], Pain scores (FLACC) [25]), cumulative opioid/benzodiazepine dose per day; and ventilator settings(ventilator rate (vent RR), positive end-expiratory pressure (PEEP) (Additional file 2)).

Analyses were performed with EZR (Saitama Medical Center, Jichi Medical University, Saitama, Japan) and R (R Core Team, Vienna, Austria). Additional details on the materials and methods are provided in the Additional file 2.

## Results

**Fig. 2:**
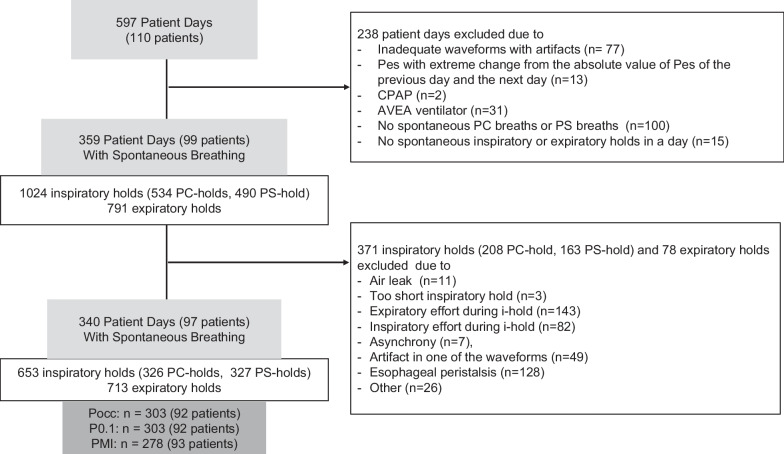
Flow chart. 110 patients with 597 total days of ventilation for whom pressure-flow waveform data was available. 238 examination days were excluded from analysis. In addition, 371 inspiratory holds (208 PC-hold, 163 PS-hold) and 78 expiratory holds met exclusion criteria, leaving 97 patients, 340 patient days, 653 inspiratory holds and 713 expiratory holds for analysis. From these, 303 Pocc, 303 P0.1, 278 PMI, and 340 ∆Pes, the median values recorded from a maximum of three breaths or holds on the same patient day, were included in the analysis

The REDvent study is ongoing and has enrolled 212 patients to date. For this analysis, we evaluated data from a sample of 110 patients with 597 total days of ventilation for whom pressure-flow waveform data were available. Of these, 238 examination days were excluded from analysis (most common reasons no spontaneous effort, AVEA ventilator, significant artifacts in the Paw or Pes Waveform) (Fig. 2). In addition, 371 of 1024 inspiratory holds (208 PC-hold, 163 PS-hold) and 78 of 791 expiratory holds met exclusion criteria, leaving 97 patients, 340 patient days, 653 inspiratory holds and 713 expiratory holds for analysis (Fig. 2). From these, the median values for each variable recorded from a maximum of three breaths or holds on the same patient day were included in the analysis, 303 Pocc, 303 P0.1, 278 PMI, and 340 ∆Pes (Fig. 2).

Patient characteristics and daily clinical parameters stratified by age are shown in Table 1 and 2. Pre-existing pulmonary (36%) or neurologic disease (38%) were the most common co-morbidities (Table 1). When stratifying by the three age groups (infant, child, adolescent) there were differences in Body Mass Index (BMI), presence of home respiratory support, F_I_O_2_, PEEP, and ventilator rate (vent RR) (Tables 1, 2). Peak flow, resistance, and actual RR also differed significantly by age group, as did sedation and pain scale scores, with younger age groups tending to be less sedated with higher pain scores (Table 2). When stratified by ventilator mode, peak pressure and delta airway pressure (Ppeak—PEEP) were higher in SIMV than PSV, while ∆Pes was greater in PSV than in SIMV. In addition, Pocc and PMI were greater in patients on PSV mode, while P0.1 was not different by ventilator mode. (Additional file 2: Table E1).

**Table 1:** Patient characteristics stratified by age

	ALL (*N* = 97)	< 1 year (*N* = 16)	1 year to < 9 years (*N* = 50)	> = 9 years to < = 18 years (*N* = 31)	*p*
Age (years)	4.3 (1.6, 12.1)	0.5 (0.4, 0.7)	3.1 (2.2, 5.6)	15.3 (12.4, 17.1)	N/A
Gender (male, %)	45 (46.4%)	6 (37.5%)	27 (54.0%)	12 (38.7%)	0.3
Weight (kg)	16.6 (10.0, 40.0)	6.4 (6.1, 7.4)^a,b^	13.7 (10.5, 18.7)^a,c^	54.2 (40.7, 68.0)^b,c^	< 0.001
Height (cm)	100 (76.0, 137.0)	63.5 (60.0, 66.4)^a,b^	90 (79.5, 105.8)^a,c^	153 (138.0, 164.3)^b,c^	< 0.001
BMI (kg/m^2^)	18.0 (15.7, 22.1)	16.5 (14.9, 18.5)^b^	16.6 (15.2, 19.5)^c^	22.6 (19.3, 28.5)^b,c^	< 0.001
Chronic pulmonary disease (n, %)	35 (36.1%)	4 (25%)	22 (44%)	9 (29%)	0.237
Home oxygen or NIV, or IMV (n, %)	17 (17.5%)	1 (6.2%)	16 (32.0%)^c^	0 (0.0%)^c^	< 0.001
Chronic neurological/neuromuscular disease (n, %)	37 (38.1%)	2(12.5%)	24 (48%)	11 (35.5%)	0.037

^a^Significant difference between < 1 year and 1 year to < 9 years

^b^Significant difference between < 1 year and >  = 9 years to <  = 18 years

^c^Significant difference between 1 year to < 9 years and >  = 9 years to <  = 18 years

**Table 2:** Daily clinical parameters stratified by age

	ALL (*N* = 340)	< 1 year (*N* = 55)	1 year to < 9 years (*N* = 185)	> = 9 years to < = 18 years (*N* = 100)	*p*
**Ventilator settings**					
Ventilator mode (SIMV, %)	200 (58.8%)	35 (63.6%)	106 (57.2%)	59 (59.0%)	0.703
F_I_O_2_	0.40 (0.35, 0.50)	0.35 (0.30, 0.40)^b^	0.40 (0.35, 0.50)^c^	0.40 (0.35, 0.55)^b, c^	0.009
PEEP (cmH_2_O)	8.2 (6.1, 10.3)	6.6 (6.1, 8.3)^b^	8.1 (6.0, 10.2)^c^	10.0 (8.0, 12.2)^b,c^	0.001
Peak pressure (cmH_2_O)	18.6 (15.7, 22.9)	18.6 (14.4, 22.5)	18.5 (15.9, 22.8)	19.8 (15.8, 23.2)	0.555
Ppeak—PEEP (cmH_2_O)	11.0 (8.5, 13.2)	12.2 (7.9, 14.5)	11 (8.8, 13.6)	10.4 (8.3, 12.7)	0.276
Vent RR (/min)	12 (0, 18)	13.5 (0, 22)	14 (0, 20)^c^	10 (0, 15)^c^	< 0.001
**Respiratory mechanics**					
Peak flow (L/s)	0.27 (0.19, 0.44)	0.15 (0.12, 0.17)^a,b^	0.25 (0.20, 0.33)^a,c^	0.56 (0.39, 0.70)^b,c^	< 0.001
Resistance (cmH_2_O/L/s)	62 (32, 97)	139 (98, 173)^a,b^	69 (44, 94)^a,c^	27 (21, 36)^b,c^	< 0.001
Actual RR (/min)	30 (23,36)	36.5 (30, 47.5)^a,b^	30 (25, 35)^a,c^	23 (18, 30)^b,c^	< 0.001
C_RS_/PBW (mL/cmH_2_O/kg)	0.64 (0.50, 0.90) *n* = 243	0.61 (0.47, 0.74) *n* = 35	0.70 (0.54, 0.95) *n* = 135	0.58 (0.48, 0.83) *n* = 73	0.533
C_CW_/PBW (mL/cmH_2_O/kg)	4.4 (2.8, 7.2) *n* = 243	3.7 (2.7, 5.8) *n* = 35	5.3 (3.2, 8.2) *n* = 135	3.5 (2.4, 5.8) *n* = 73	0.426
E_L_/E_RS_	0.84 (0.77, 0.89) *n* = 243	0.85 (0.78, 0.87) *n* = 35	0.84 (0.78, 0.90) *n* = 135	0.83 (0.74, 0.88) *n* = 73	0.589
∆Pes (cmH_2_O)	8.5(5.2, 12.3)	7.4 (3.9, 10.8)	9.3 (6.0, 13.8)	7.5 (4.5, 12.2)	0.309
Pocc (cmH_2_O)	13.2 (8.9, 18.3) *n* = 303	11.3 (8.3, 14.5) *n* = 50	13.7 (9.3, 18.9) *n* = 168	12.8 (8.6, 18.5) *n* = 85	0.268
P0.1 (cmH_2_O)	0.8 (0.5, 1.6) *n* = 303	0.8 (0.5, 1.4) *n* = 50	0.8 (0.5, 1.6) *n* = 168	0.8 (0.5, 1.6) *n* = 85	0.615
PMI (cmH_2_O)	1.5 (− 0.5, 4.3) *n* = 278	1.0 (− 0.8, 3.3) *n* = 43	1.4 (− 0.4, 3.8) *n* = 154	1.8 (− 0.5, 4.9) *n* = 81	0.358
**Gas exchange**					
pH	7.41 (7.37, 7.43) *n* = 339	7.40 (7.37, 7.43) *n* = 54	7.41 (7.37, 7.43) *n* = 185	7.42 (7.38, 7.44) *n* = 100	0.652
PCO_2_ (mmHg)	45 (40, 51) *n* = 339	46 (40, 50) *n* = 54	46 (41, 51) *n* = 185	43 (39, 51) *n* = 100	0.609
**Pain/sedation**					
Opioid/PBW (ug/kg/day)	106 (2, 2644)	753 (2, 3508)	109 (3, 2740)	10 (1, 2467)	0.957
Benzo/PBW (mg/kg/day)	0.8 (0, 2.6)	1.6 (0, 3.5)	0.9 (0, 2.7)	0 (0, 1.5)	0.061
FLACC	0.5 (0, 2)	2 (1, 3)^a,b^	1 (0,2)^a,c^	0 (0, 1)^b,c^	< 0.001
SBS	− 1 (− 1, 0)	0 (− 1, 0)^a,b^	− 1 (− 1, 0)^a,c^	− 1 (− 1, 0)^b,c^	0.002

^a^Significant difference between < 1 year and 1 year to < 9 years

^b^Significant difference between < 1 year and >  = 9 years to <  = 18 years

^c^Significant difference between 1 year to < 9 years and >  = 9 years to <  = 18 years

### Comparison of correlations between ∆Pes and Pocc/P0.1/PMI

**Fig. 3:**
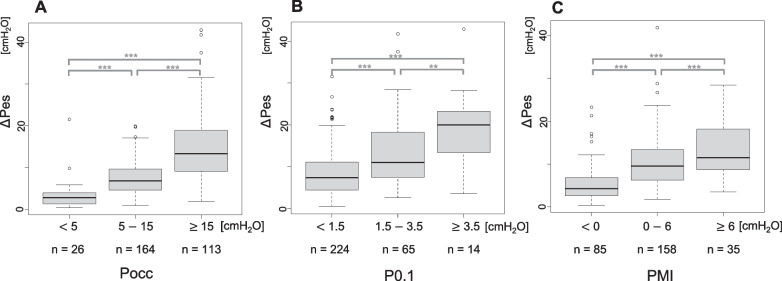
Comparison of ∆Pes by group based on thresholds for Pocc (**A**), P0.1 (**B**) and PMI (**C**). There is a dose response relationship with all variables and ∆Pes, although there is more overlap in ranges for P0.1 and PMI than Pocc. Median (bar), interquartile range (box), non-outlier range (whiskers). The thresholds separating each group were set with reference to studies of adults (Pocc, P0.1, PMI) and children (PMI) [9, 21, 33]. Significant differences are shown with the ***(*p* < 0.001), ** (*p* <  0.01) based on linear mixed modeling to control for repeated measures

Using all data points with repeated measures analysis, Pocc had the strongest correlation with ∆Pes *r* = 0.68 (95% confidence interval (CI): 0.59, 0.74), followed by PMI with 0.60 (95% CI 0.50, 0.69) and then P0.1 with 0.42 (95% CI 0.31, 0.53) (Table 3, Additional file 1: Figure E1, E2). There is a dose–response relationship between Pocc/P0.1/PMI and ∆Pes (Fig. 3) using thresholds suggested in previous studies [5, 10], however, P0.1 and PMI had a greater overlap between groups compared to Pocc. Limiting analysis to one random observation per patient to derive regression equations (Table 4) again confirmed that Pocc most reliably predicted ∆Pes and can be given by the equation ∆Pes = 0.66 × Pocc. The respective thresholds for detecting high and low effort based on ∆Pes cut points using the regression equations, as well as the sensitivity and specificity are shown in Table 5.

**Table 3:** Correlation coefficients between ∆Pes and Pocc/P0.1/PMI

	ALL (*N* = 340)	< 1 year (*N* = 55)	1 year to < 9 years (*N* = 185)	> = 9 years to < = 18 years (*N* = 100)
Pocc (cmH_2_O)	*r* = 0.68 (95% CI 0.59, 0.74)	*r* = 0.80 (95% CI 0.70, 0.90)	*r* = 0.63 (95% CI 0.52, 0.70)	*r* = 0.78 (95% CI 0.72, 0.89)
P0.1 (cmH_2_O)	*r* = 0.42 (95% CI 0.31, 0.53)	*r* = 0.53 (95% CI 0.22, 0.66)	*r* = 0.45 (95% CI 0.27, 0.59)	*r* = 0.25 (95% CI − 0.05, 0.58)
PMI (cmH_2_O)	*r* = 0.60 (95% CI 0.50, 0.69)	*r* = 0.60 (95% CI 0.354, 0.80)	*r* = 0.61 (95% CI 0.44, 0.75)	*r* = 0.61 (95% CI 0.43, 0.76)

**Table 4:** Regression equations based on one random sample per patient for the relationship between delta Pes and Pocc, P0.1, PMI

	Intercept	Coefficient (Beta, 95% CI)	Regression equation	*R*^2^
Pocc	0*	0.66 (95% CI 0.60, 0.73)	∆Pes = 0.66 $\times$ Pocc	0.82
P0.1	0*	5.91 (95% CI 5.04, 6.78)	∆Pes = 5.91 $\times$ P0.1	0.68
PMI	7.5	1.20 (95% CI 0.79,1.60)	∆Pes = 7.5 + 1.20 $\times$ PMI	0.31

*Intercept forced through zero for Pocc and P0.1 because both parameters should be zero when ∆Pes is zero

**Table 5:** The estimated thresholds, sensitivity and specificity for detecting high and low inspiratory effort

	ΔPes < 3 *N* = 14	ΔPes < 5 *N* = 22	ΔPes > 12 *N* = 27	ΔPes > 15 *N* = 17	ΔPes > 20 *N* = 11
Pocc (cmH_2_O)	< 4.5 *N* = 7	< 7.5 *N* = 17	> 17.9 *N* = 30	> 22.4 *N* = 12	> 29.9 *N* = 7
	Sens: 0.33	Sens: 0.60	Sens: 0.80	Sens: 0.63	Sens: 0.45
	Spec: 0.96	Spec: 0.93	Spec: 0.84	Spec: 0.93	Spec: 0.97
P 0.1 (cmH_2_O)	< 0.5 *N* = 27	< 0.9 *N* = 39	> 2.0 *N* = 17	> 2.5 *N* = 12	> 3.4 *N* = 5
	Sens: 0.50	Sens: 0.65	Sens: 0.52	Sens: 0.63	Sens: 0.36
	Spec: 0.72	Spec: 0.61	Spec: 0.94	Spec: 0.97	Spec: 0.99
PMI (cmH_2_O)	< − 3.8 *N* = 3	< − 0.4 *N* = 9	> 3.8 *N* = 23	> 6.3 *N* = 7	> 10.4 *N* = 1
	Sens: 0.13	Sens: 0.30	Sens: 0.68	Sens: 0.17	Sens: 0.0
	Spec: 0.99	Spec: 0.95	Spec: 0.86	Spec: 0.93	Spec: 0.99

### Subgroup analysis

In subgroup analysis limited to patients on PSV, correlations were slightly weaker between all parameters and ∆Pes, although Pocc retained the strongest correlation with ∆Pes (*r* = 0.59 (95% CI 0.46, 0.67)) followed by P0.1 (*r* = 0.48 (95% CI 0.18, 0.68)) and PMI (*r* = 0.45 (95% CI 0.24, 0.63)) (Additional file 2: Table E2). As with the results for patients on all ventilator modes, there is a dose–response relationship between Pocc/P0.1/PMI and ∆Pes when restricted to patients on PSV (Additional file 1: Figure E3). When stratifying by age, the dose–response relationship between Pocc/P0.1/PMI and ∆Pes is consistent within all age groups although the number of patients in some of the strata are low (Additional file 1: Figure E4). Pocc maintained a high correlation with ∆Pes, regardless of age group, and outperformed both PMI and P0.1 in all age groups (Table 3). There were some age-related differences, with P0.1 having a weaker correlation with ∆Pes in older age patients, and PMI having a weaker correlation with ∆Pes in PSV in younger patients (Additional file 2: Table E2).

### Sensitivity analyses

Age related subgroup differences were less pronounced when restricting to the 144 test days with the most optimal calibration of the esophageal catheter during the occlusion test (OT) (Additional file 2: Sensitivity Analysis 1, Table 3, Additional file 2: Tables E2, E3). In addition, results did not differ significantly when using Pmusc as the measure for respiratory effort as compared to delta Pes (Additional file 2: Sensitivity analysis 2). Finally, in SIMV mode, delta Pes did not vary significantly based on whether the breath was PC or PS (Additional file 2: Sensitivity Analysis 3, Additional file 1: Figure E5).

## Discussion

We have shown the feasibility and accuracy of using airway occlusion maneuvers to estimate respiratory effort in mechanically ventilated children with ARDS. Overall, we found Pocc, measured as the net negative deflection in airway pressure during inspiratory effort following an end-expiratory occlusion, showed the best correlation with esophageal pressure in all age groups and ventilation modes. PMI and P0.1 had reasonable correlations with esophageal pressure, but there was some variability as a function of age and mode of ventilation. Overall, the occlusion maneuvers that were conducted during expiratory holds (Pocc and P0.1) had a lower failure rate than PMI (inspiratory hold) and could be calculated on many more patients than PMI, which requires the patient to become passive during end-inspiration. We believe these findings support using Pocc more systematically to estimate respiratory effort amongst ventilated children, including potential use in clinical trials which require a measure of respiratory effort. We believe that using the equation ∆Pes = 0.66 × Pocc (Table 4) would likely be appropriate in children, and, surprisingly, is completely consistent with adult studies, which propose the equation as ∆Pes = 2/3 × Pocc [12]. Pocc values also have reasonable sensitivity and excellent specificity to detect ∆Pes values which may be therapeutic targets for high or low respiratory effort.

There are multiple calculations which can be done with esophageal manometry to estimate patient effort, including work of breathing (which integrates esophageal pressure and spirometry and may incorporate respiratory rate), effort of breathing (pressure–time product, pressure rate product), and ∆Pes (peak to trough change in esophageal pressure) [4, 26, 27]. These parameters are also sometimes adjusted for chest wall elastance. While these markers remain the gold standard, they are difficult to operationalize in routine clinical practice. Alternative measures of airway pressure during end-expiratory (P0.1, Pocc) and end-inspiratory occlusions (PMI) have been proposed [12, 14, 17], but ours is the first study to evaluate these three maneuvers comprehensively and simultaneously, particularly in children. We chose to validate them against ∆Pes rather than work or effort of breathing parameters, as all are measures of effort on a single breath. Pmusc takes chest wall elastance into account and evaluates inspiratory effort in a single breath, but its calculation requires C_CW_, which needs complete relaxation of respiratory muscles during an inspiratory hold. This limits the number of cases in which it can be measured in spontaneously breathing patients, and systematically eliminates patients who have inspiratory effort during an inspiratory hold (which are often the patients with the highest values for delta Pes). Nevertheless, the correlation between Pmusc and delta Pes was almost perfect (0.97, Additional file 2: Sensitivity Analysis 2), hence we believe it to be appropriate to use ΔPes rather than Pmusc for the present comparison, to ensure we can use measurements from a more representative sample of patients.

These three airway occlusion parameters are related to one another but do represent different physiologic concepts. We found that Pocc had the strongest correlation with ∆Pes, which is expected. During airway occlusion, the total change in airway pressure should closely estimate the total change in esophageal pressure [4]. Moreover, if the occlusion is brief (1–2 s), then the respiratory pattern from a previous un-occluded to an occluded breath is not expected to change significantly, and Pocc would be expected to reflect ∆Pes of un-occluded breaths [12]. Theoretically, Pocc and ∆Pes should capture both resistive and elastic components of respiratory effort. Pocc retained the strongest association with ∆Pes across all age groups and ventilator modes.

P0.1 reflects the change in airway pressure during the first 100 ms of an inspiratory effort and is thought to reflect both respiratory effort and drive [14, 28]. We found that the relationship between P0.1 and ∆Pes differed as a function of age, and was stronger for young patients, especially on PS ventilation, and weaker for older patients. This may relate to relatively shorter inspiratory times for infants on PS. There was a dose–response relationship between P0.1 and ∆Pes, however, the range of values is relatively narrow and we found it was difficult to identify P0.1 thresholds which would reliably discriminate between high or low inspiratory effort. This may be influenced by age-related differences in inspiratory time, as described above.

PMI is calculated as the difference between plateau pressure and peak pressure during an end-inspiratory hold, and primarily reflects the elastic component of the respiratory system [15]. It makes sense, then, that the correlation between ΔPes and PMI is inferior to the correlation of ΔPes with Pocc, which also takes the resistive component into account. The correlation between PMI and ∆Pes was weaker on pressure support ventilation, particularly for younger children. This may relate to higher respiratory drive and shorter inspiratory times which may affect the inspiratory hold.

There are several limitations to this study. First, it is primarily a physiologic study to evaluate the relationship between these parameters and ∆Pes. Hence, we do not know whether these parameters will be useful in clinical practice as we did not evaluate any relationship between these parameters and outcome, although that is a logical next step. Second, esophageal balloon calibration was conducted prior to measurements [4, 29, 30], but there were still patients in whom the expected calibration ratio between airway pressure and esophageal pressure of 0.8–1.2 was not achieved. We have found this to be not uncommon, particularly in younger children [31], and have expanded on this analysis in the Additional file 2. This may relate to inherent limitations of air-filled balloon catheters when patients have rapid reductions in pleural pressure as occurs during airway occlusion. Baseline characteristics of the patients who met and did not meet the criteria of the expected calibration ratio were different, and most notably related to chest wall versus lung elastance. Excluding these patients from the analysis may exclude certain populations such as those with good Ccw. Nevertheless, sensitivity analysis restricting it to measurements which met this calibration requirement did not substantially change the results. Third, we based our analysis on ∆Pes, not Pmusc, which is the gold standard estimate of patient effort because it also considers the contribution from the chest wall. Unfortunately, calculation of Pmusc does require an estimate of Ccw, which requires the patient to become passive at end-inspiration. As we have shown this will select against some types of patients, limiting generalizability. Nevertheless, our sensitivity analysis (Additional file 2) confirmed very strong correlation between Pmusc and Delta Pes (0.97). Fourth, ∆Pes was measured during several breaths before and after the occlusion maneuver, and it is possible that in some children, respiratory patterns may have changed just prior to the occlusion maneuver. This could affect the correlation. Fifth, we post processed all waveforms, and did not use the ventilator screens themselves (or built-in P0.1 measurements) to compute the values used in this study. Previous work has highlighted that P0.1 obtained from most commercially available ventilators are generally accurate compared to post-processed measures [32]. Pocc and PMI are not standard parameters in most ventilators but can be computed by freezing the screen during either inspiratory or expiratory holds on most ventilators (Additional file 1: Figure E6). However, there are some ventilators which are programmed to automatically open the valve when spontaneous breathing is sensed during inspiratory or expiratory occlusion, which would invalidate these measurements.

## Conclusions

Pocc, P0.1, and PMI, which are measures of airway pressure obtained from inspiratory and expiratory holds, may all be useful alternatives to esophageal pressure to estimate the magnitude of inspiratory effort in ventilated children. Pocc represents the most promising target, as it has the strongest correlation with ∆Pes for all age groups and modes of ventilation and can be readily measured on nearly all patients.

### Supplementary Information

**Additional file 1. Figure E1**: Scatter plots for the repeated measure correlations between Pocc (A), P0.1 (B), PMI (C) and ∆Pes using cube-transformed ∆Pes and log-transformed P0.1. r: repeated measure correlation using cube-transformed ∆Pes and log-transformed P0.1 [95% CI]. Pocc showed the strongest correlation with ∆Pes, followed by PMI and P0.1. **Figure E2**: Scatter plots for the repeated measure correlations between Pocc (A), P0.1 (B), PMI (C) and ∆Pes using non-transformed measures. r: repeated measure correlation using non-transformed data [95% CI]. As with Figure E1, Pocc showed the strongest correlation with ∆Pes, followed by PMI and P0.1. **Figure E3**: Comparison of ∆Pes by groups for each threshold of Pocc (A), P0.1 (B) and PMI (c) in PSV-mode. Significant differences are shown with the *** (p < 0.001), *(P<0.05) based on linear mixed modeling to control for repeated measures. Similar to the results for all patients, there is a dose-response relationship in the PSV mode for almost all variables and ∆Pes, but the overlap in range is greater for P0.1 and PMI than for Pocc. Median (bar), interquartile range (box), non-outlier range (whiskers). **Figure E4**: Comparison of ∆Pes by groups for each threshold of Pocc , P0.1 and PMI in different age groups. Median (bar), interquartile range (box), non-outlier range (whiskers). Significant differences are shown with the *** (p < 0.001), **(P<0.01), *(P<0.05) based on linear mixed modeling to control for repeated measures. For all age groups, there is a dose-response relationship in the PSV mode for almost all variables and ∆Pes, but the overlap in range is greater for P0.1 and PMI than for Pocc. **Figure E5**: The Bland-Altman analysis for the agreement of ∆Pes between PC and PS breath in each same patient in SIMV mode. ∆Pes in PC and PS breaths were about the same level. **Figure E6**: Example of Pocc (A) and PMI (B) measurement using a commercially available ventilator. Pocc and PMI can be measured by using the expiratory (Pocc) or inspiratory (PMI) hold function on the ventilator and freezing the screen. Pocc is calculated as the difference in the pressure from PEEP to the most negative point during the inspiratory attempt (Paw(trough)). PMI is calculated as the difference between Plateau Pressure and Peak Pressure. NKV-550 (OrangeMed, Santa Ana, CA) was used here.

**Additional file 2. Table E1**: Daily clinical parameters stratified by ventilator mode. **Table E2**: Correlation coefficient between ∆Pes and Pocc/P0.1/PMI on PSV mode. **Table E3**: Correlation coefficient between ∆Pes and Pocc/P0.1/PMI using patient days with delta Pes/delta Paw 0.8 to 1.2 in end-expiratory occlusion.

## Data Availability

The datasets generated and/or analyzed during the current study are not publicly available due to the ongoing nature of the clinical trial, but are available from the corresponding author on reasonable request.

## Abbreviations

∆Pes: Esophageal pressure during tidal breathing,
Pocc: Expiratory occlusion pressure
P0.1: Airway occlusion pressure
PMI: Respiratory muscle pressure index
ARDS: Acute respiratory distress syndrome
VIDD: Ventilator-induced diaphragm dysfunction
P-SILI: Patient self-inflicted lung injury
PC: Pressure control
PS: Pressure support
Pes: Esophageal pressure
Paw: Airway pressure
SIMV: Synchronized Intermittent Mandatory Ventilation
PSV: Pressure Support Ventilation
Pmusc: Respiratory muscle pressure
C_RS_: Static respiratory system compliance
C_CW_: Chest wall compliance
E_L_: Lung elastance
E_RS_: Respiratory system elastance
actual RR: Respiratory rate
SBS: State Behavioral Scale
vent RR: Ventilator rate
PEEP: Positive end-expiratory pressure
BMI: Body Mass Index
Ppeak: Peak pressure
CI: Confidence interval
PBW: Predicted body weight
