# Supplementary material for: Estimation of inspiratory effort using airway occlusion maneuvers in ventilated children: a secondary analysis of an ongoing randomized trial testing a lung and diaphragm protective ventilation strategy
Source: Crit Care. 2023 Nov 29;27:466. doi: 10.1186/s13054-023-04754-6 (PMC10685539; doi:10.1186/s13054-023-04754-6)
Supplement: Supplementary file 1 — Additional file 1. Figure E1: Scatter plots for the repeated measure correlations between Pocc (A), P0.1 (B), PMI (C) and ∆Pes using cube-transformed ∆Pes and log-transformed P0.1. r: repeated measure correlation using cube-transformed ∆Pes and log-transformed P0.1 [95% CI]. Pocc showed the strongest correlation with ∆Pes, followed by PMI and P0.1. Figure E2: Scatter plots for the repeated measure correlations between Pocc (A), P0.1 (B), PMI (C) and ∆Pes using non-transformed measures. r: repeated measure correlation using non-transformed data [95% CI]. As with Figure E1, Pocc showed the strongest correlation with ∆Pes, followed by PMI and P0.1. Figure E3: Comparison of ∆Pes by groups for each threshold of Pocc (A), P0.1 (B) and PMI (c) in PSV-mode. Significant differences are shown with the *** (p < 0.001), *(P<0.05) based on linear mixed modeling to control for repeated measures. Similar to the results for all patients, there is a dose-response relationship in the PSV mode for almost all variables and ∆Pes, but the overlap in range is greater for P0.1 and PMI than for Pocc. Median (bar), interquartile range (box), non-outlier range (whiskers). Figure E4: Comparison of ∆Pes by groups for each threshold of Pocc , P0.1 and PMI in different age groups. Median (bar), interquartile range (box), non-outlier range (whiskers). Significant differences are shown with the *** (p < 0.001), **(P<0.01), *(P<0.05) based on linear mixed modeling to control for repeated measures. For all age groups, there is a dose-response relationship in the PSV mode for almost all variables and ∆Pes, but the overlap in range is greater for P0.1 and PMI than for Pocc. Figure E5: The Bland-Altman analysis for the agreement of ∆Pes between PC and PS breath in each same patient in SIMV mode. ∆Pes in PC and PS breaths were about the same level. Figure E6: Example of Pocc (A) and PMI (B) measurement using a commercially available ventilator. Pocc and PMI can be measured by using the expiratory (Pocc) [file 13054_2023_4754_MOESM1_ESM.pptx]

## Slide 1
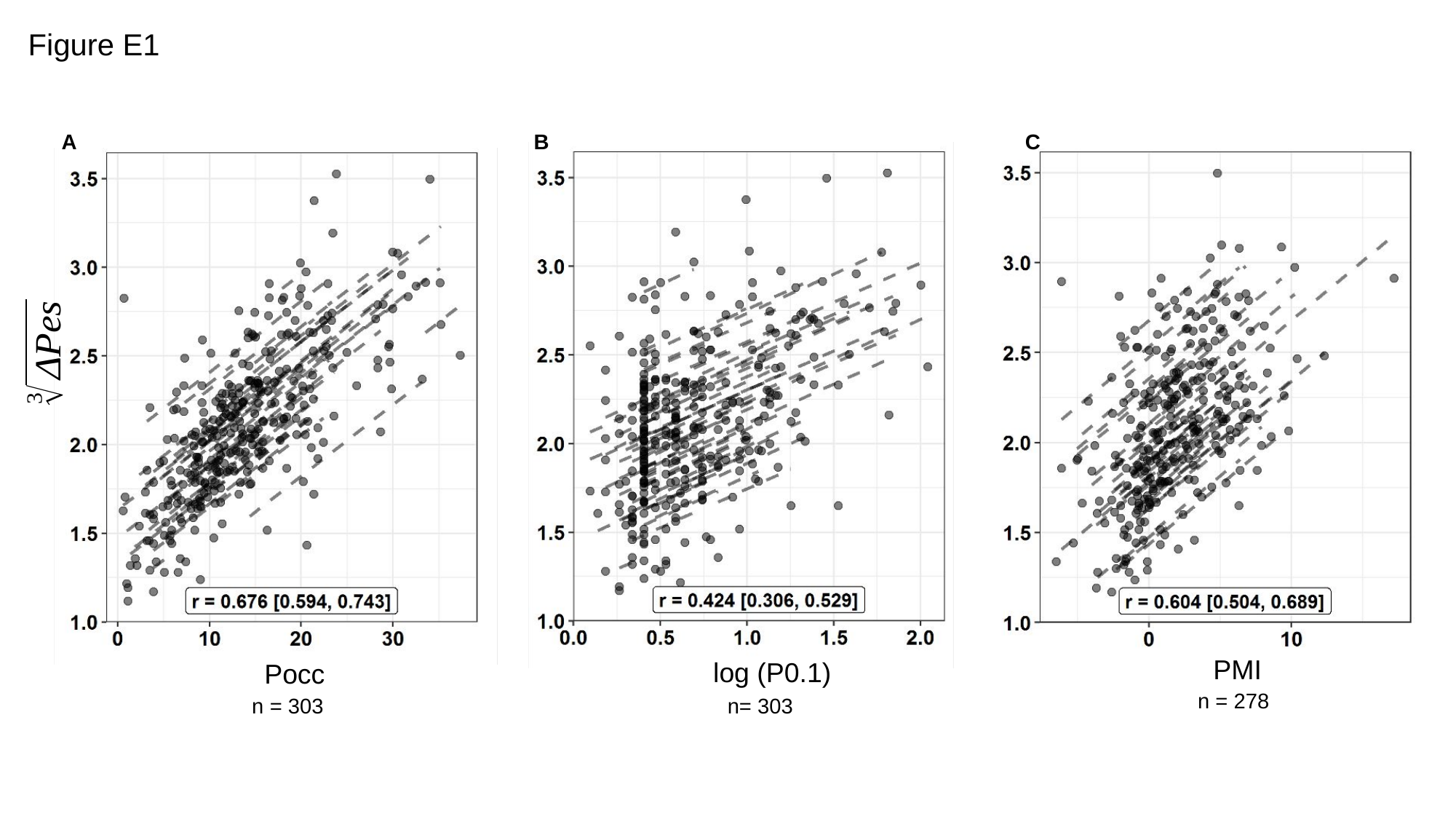

Figure E1
A
B
C
PMI
log (P0.1)
Pocc
n = 278
n= 303
n = 303

## Slide 2
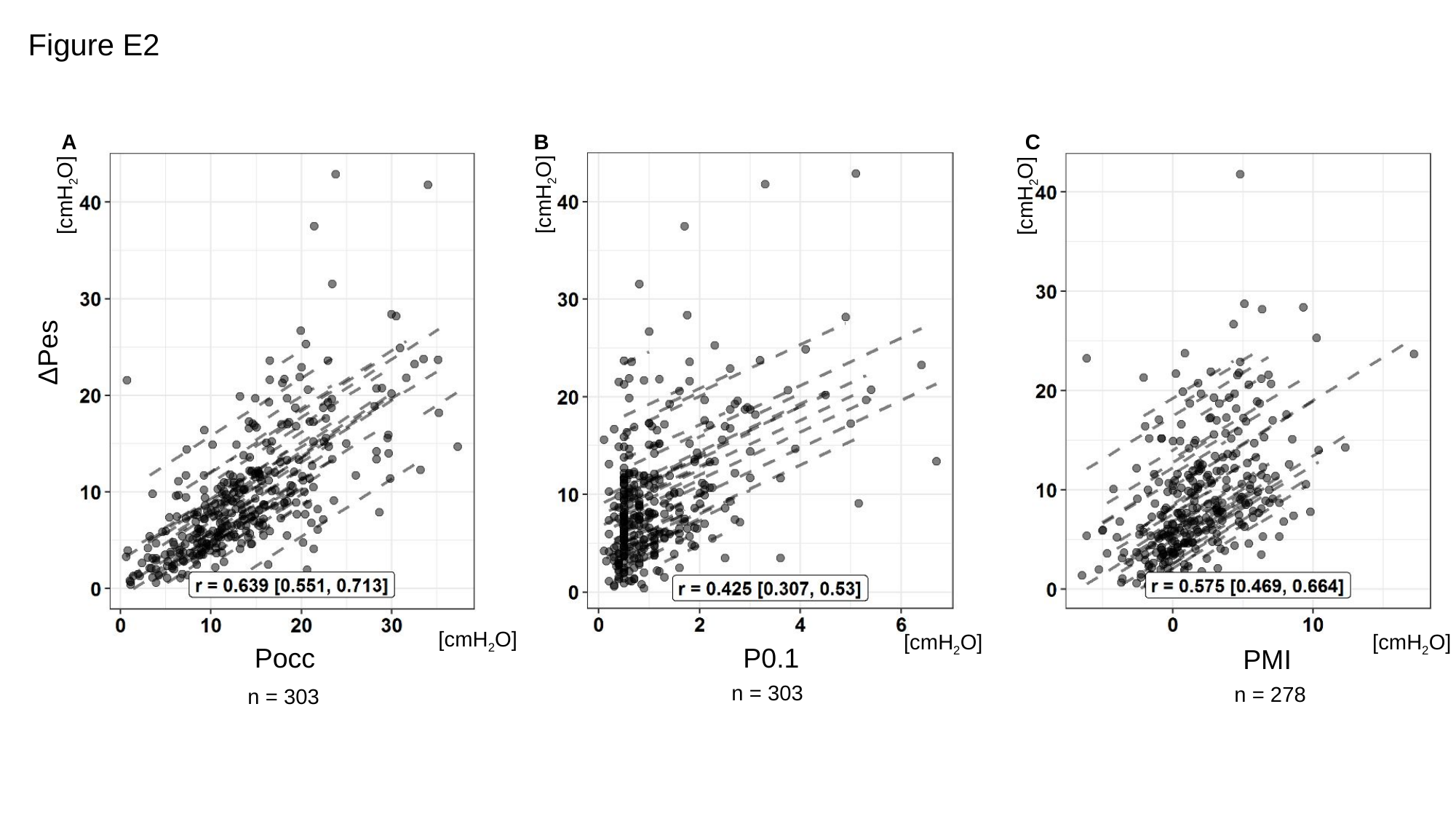

Figure E2
A
B
C
[cmH2O]
[cmH2O]
[cmH2O]
ΔPes
[cmH2O]
[cmH2O]
[cmH2O]
P0.1
Pocc
PMI
n = 303
n = 278
n = 303

## Slide 3
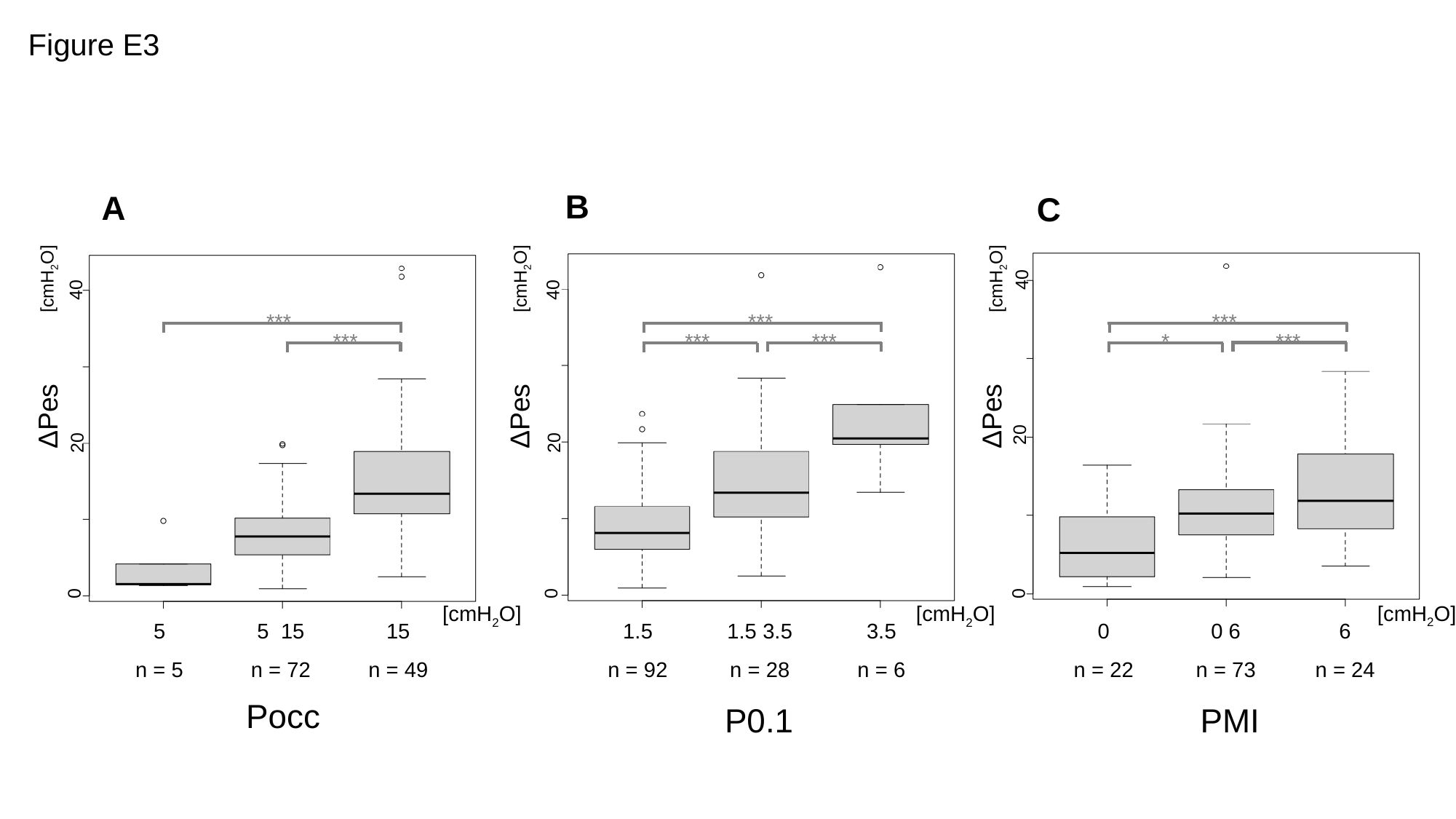

Figure E3
B
A
C
[cmH2O]
[cmH2O]
[cmH2O]
40
40
40
***
***
***
***
*
***
***
***
ΔPes
ΔPes
ΔPes
20
20
20
0
0
0
[cmH2O]
[cmH2O]
[cmH2O]
n = 5
n = 72
n = 49
n = 92
n = 28
n = 6
n = 22
n = 73
n = 24
Pocc
P0.1
PMI

## Slide 4
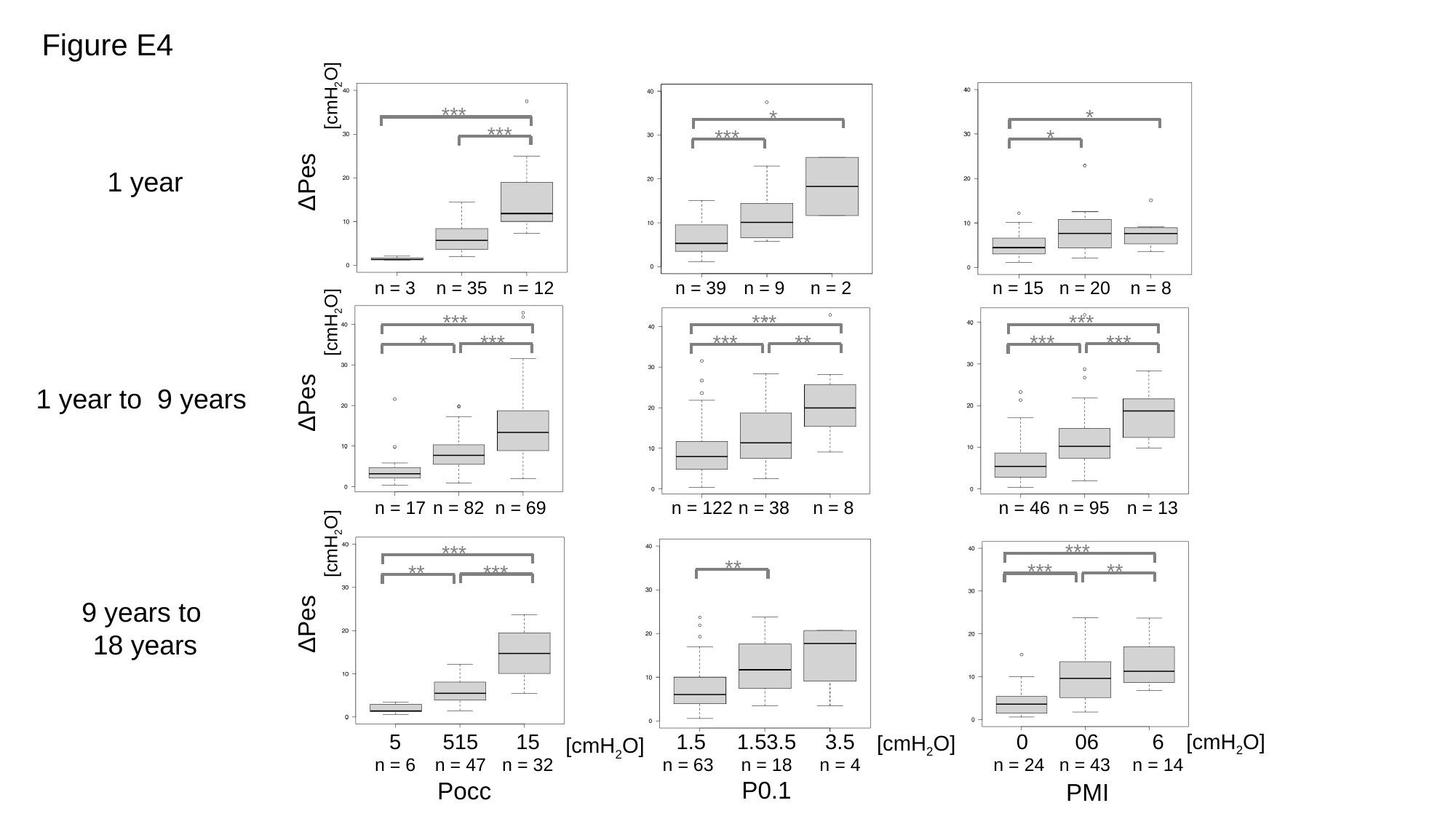

Figure E4
[cmH2O]
***
***
*
*
*
***
ΔPes
n = 3
n = 35
n = 12
n = 39
n = 9
n = 2
n = 15
n = 20
n = 8
***
***
*
***
**
***
***
***
***
[cmH2O]
ΔPes
n = 17
n = 82
n = 69
n = 122
n = 38
n = 8
n = 46
n = 95
n = 13
[cmH2O]
***
**
***
***
***
**
**
ΔPes
[cmH2O]
[cmH2O]
[cmH2O]
n = 6
n = 47
n = 32
n = 63
n = 18
n = 4
n = 24
n = 43
n = 14
P0.1
Pocc
PMI

## Slide 5
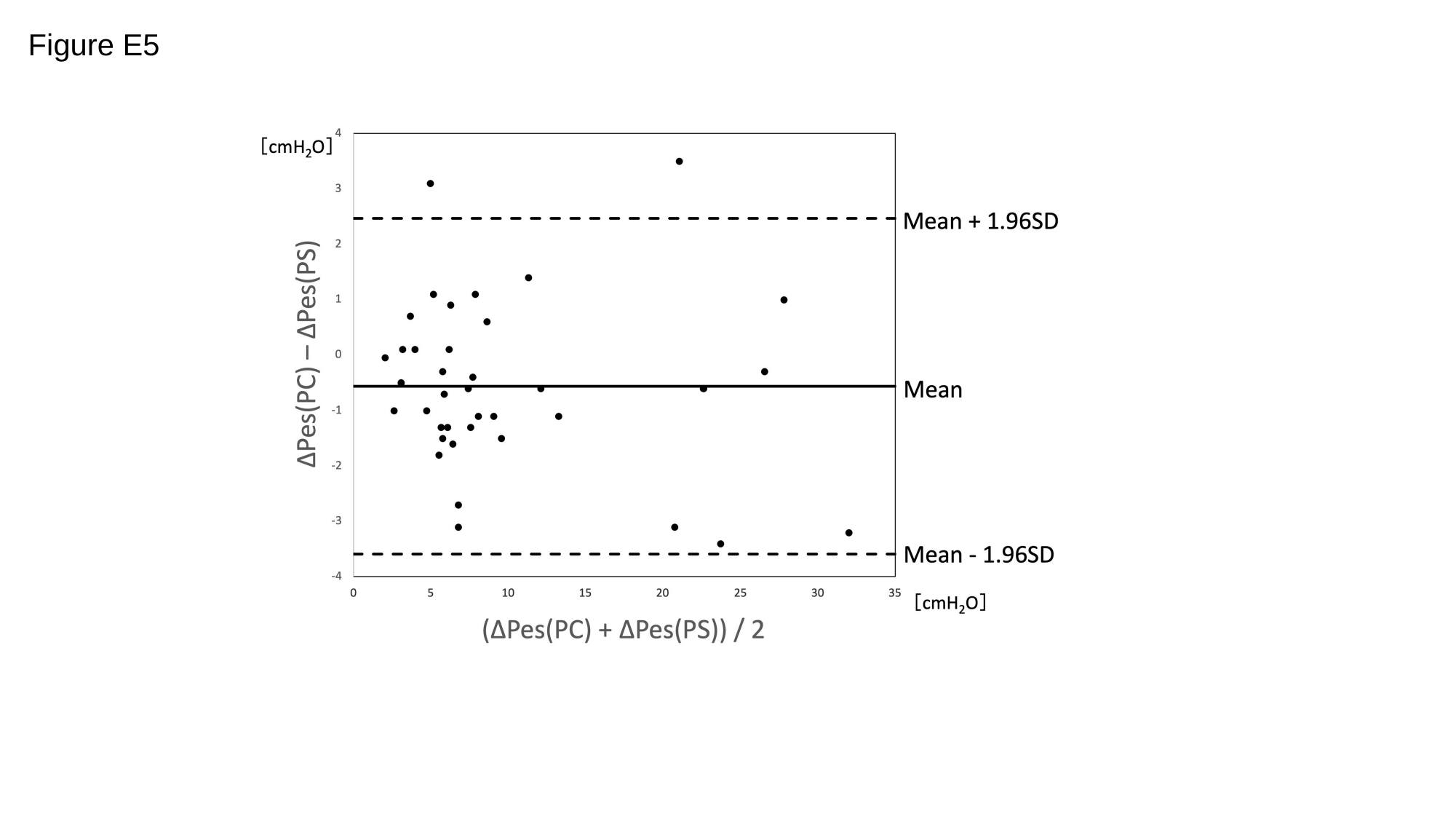

Figure E5

## Slide 6
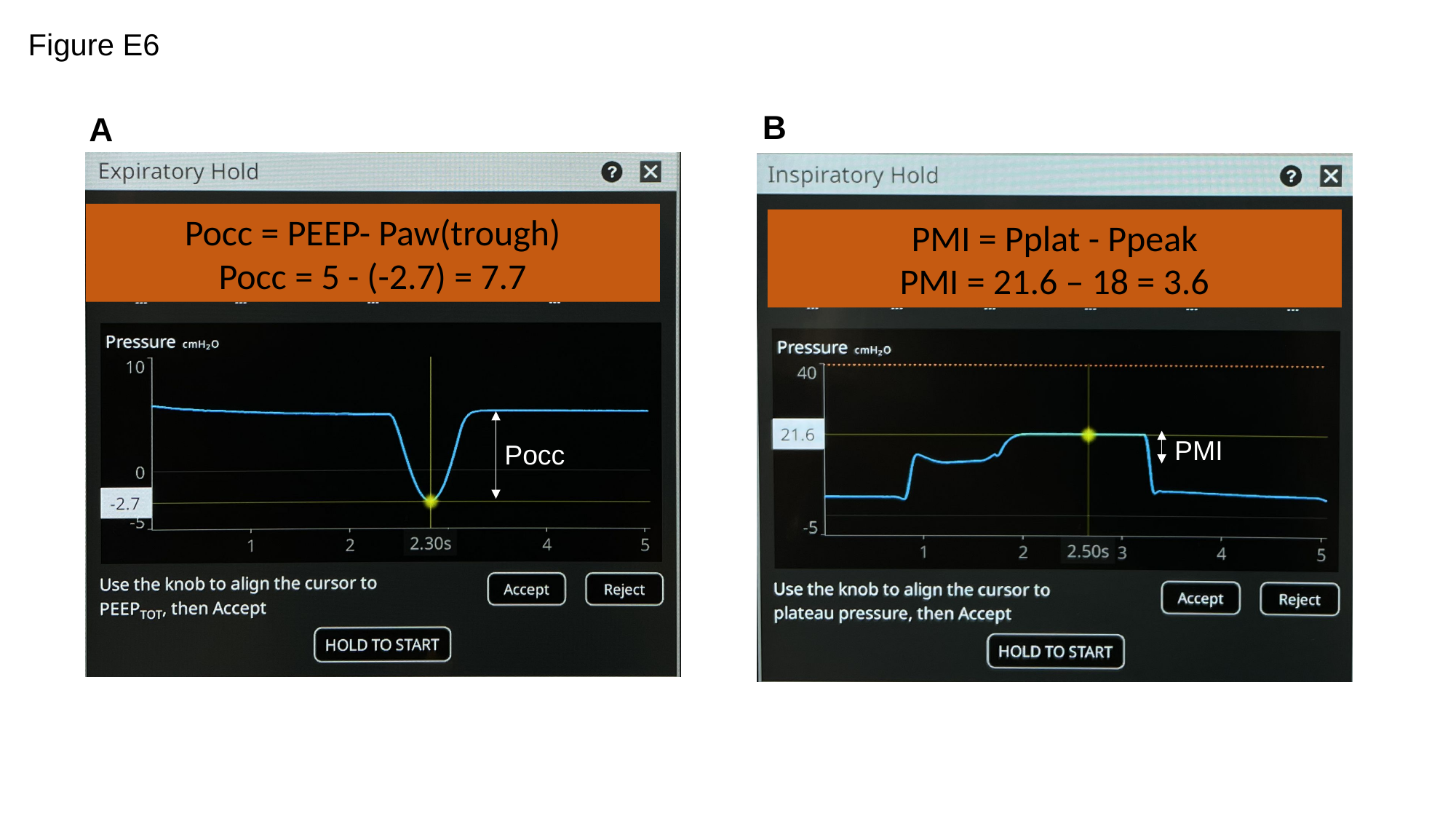

Figure E6
B
A
Pocc = PEEP- Paw(trough)
Pocc = 5 - (-2.7) = 7.7
PMI = Pplat - Ppeak
PMI = 21.6 – 18 = 3.6
PMI
Pocc
