# Supplementary material for: Estimation of inspiratory effort using airway occlusion maneuvers in ventilated children: a secondary analysis of an ongoing randomized trial testing a lung and diaphragm protective ventilation strategy
Source: Crit Care. 2023 Nov 29;27:466. doi: 10.1186/s13054-023-04754-6 (PMC10685539; doi:10.1186/s13054-023-04754-6)
Supplement: Supplementary file 2 — Additional file 2. Table E1: Daily clinical parameters stratified by ventilator mode. Table E2: Correlation coefficient between ∆Pes and Pocc/P0.1/PMI on PSV mode. Table E3: Correlation coefficient between ∆Pes and Pocc/P0.1/PMI using patient days with delta Pes/delta Paw 0.8 to 1.2 in end-expiratory occlusion. [file 13054_2023_4754_MOESM2_ESM.docx]

Estimation of Inspiratory Effort Using Airway Occlusion Maneuvers in Ventilated Children: a secondary analysis of an ongoing randomized trial testing a lung and diaphragm protective ventilation strategy.

Yukie Ito, Matías G Herrera, Justin C. Hotz, Miyako Kyogoku, Christopher J.L. Newth, Anoopindar K. Bhalla, Muneyuki Takeuchi, Robinder G. Khemani

**Online Data Supplement**

**Supplemental Materials and methods**

Patients

For the parent study, patients were excluded if they had contraindications to an esophageal catheter (i.e. severe mucosal bleeding, nasal encephalocele, trans-sphenoidal surgery), conditions precluding diaphragm ultrasound measurement (i.e. abdominal wall defects, pregnancy), conditions precluding conventional methods of weaning (i.e., status asthmaticus, severe lower airway obstruction, critical airway, intracranial hypertension, Extra Corporeal Life Support, limitation of care, severe chronic respiratory failure, spinal cord injury above lumbar region, cyanotic heart disease (unrepaired or palliated)), or cases in which the attending physician refused enrollment.

Definition of Variables

C_RS_: VT/(Pplat – PEEP)

C_CW_: VT/(Pesplat – Pespeep)

E_L_/E_RS_: C_RS_/C_CW_

Resistance: calculated as the time constant measured by the expiratory pressure-volume curve divided by C_RS_ (2).

Pmusc: ∆Pes + VT / C_CW_

C_RS_: respiratory system compliance, C_CW_: chest wall compliance, Respiratory muscle pressure (Pmusc), VT: tidal volume, PEEP: positive end-expiratory pressure, Pplat: plateau pressure, Pesplat: plateau pressure of Pes, Pespeep: end-expiratory pressure of Pes

Selection of data for Analysis

For analysis, all patients had to have evidence of spontaneous breathing, measured by negative deflection of the esophageal pressure waveform during inspiration. Data was selected for analysis at the waveform level. A maximum of three of each of the following breath types were used for each patient day: inspiratory hold (pressure control (PC) hold or pressure support (PS) hold), expiratory hold, PC breath, and PS breath. The median of each measurement per day was used in the analysis. Holds (either inspiratory or expiratory) were excluded from analysis if there was air leak greater than 20% or significant artifact in any of the waveforms. Inspiratory holds were also excluded if the patient did not become passive at end-inspiration as evidenced by a continuously rising plateau pressure (expiratory effort), negative deflection with a breath attempt (inspiratory effort), or if the hold time was inadequate (less than 0.5 seconds or no apparent plateau pressure). All recordings were reviewed by two authors, and waveforms with ambiguous calculations or exclusion criteria were discussed with other authors to reach consensus.

Analysis

Dose response relationships between Peak-to trough esophageal pressure during tidal breathing (∆Pes) and suggested cut-offs of Expiratory occlusion pressure (Pocc), airway occlusion pressure (P0.1) and respiratory muscle pressure index (PMI) are reported with box-plots. *A priori* thresholds for low and high values for each variable were chosen, adapted from previously proposed targets, using 5 and 15 cmH_2_O for Pocc, 1.5 and 3.5 cmH_2_O for P0.1, and 0 and 6 cmH_2_O for PMI (3, 4). The threshold for high PMI was set at 6 cmH_2_O because one previous study reported that a PMI of 7 cmH_2_O or higher would result in esophageal pressure (Pes) of 10 cmH_2_O or higher, but in this study there were very few observations above 7 cmH_2_O (4).

Crs, C_CW_ and E_L_/E_RS_ were adopted only if the inspiratory plateau pressure of Pes minus the expiratory plateau pressure of Pes was positive. Pmusc was calculated for breaths in the presence of C_CW_ obtained as described above. For pH and pCO_2_, measurements from arterial blood gas, venous blood gas or capillary blood gas were used; for venous blood gas, PvCO_2_ minus 5 mmHg was adopted as pCO2 (5). When appropriate, parameters were normalized to predicted body weight (i.e., tidal volume, compliance, cumulative dose of opioids) calculated by the Moore method for analysis (6).

Sensitivity Analyses

Three additional sensitivity analyses were performed to ensure the findings are representative.

Results

**Sensitivity Analysis 1: Airway Occlusion Maneuver and Balloon Calibration.**

The first sensitivity analysis focused on restricting measurements based on confirmed adequacy of balloon calibration. Previous investigations in adult patients when using esophageal manometry have used an occlusion test to ensure that the catheter is in appropriate position, and the volume of air inflated in the catheter is appropriate. When patients are spontaneously breathing, the airway is occluded during an inspiratory breath attempt, the change in airway pressure should equal the change in esophageal pressure. The accepted standard in adult patients is a delta Pes/delta Paw ratio being between 0.8 to 1.2 during the occlusion test (7–9). In children this technique has not been extensively validated in vivo, particularly when patients are spontaneously breathing. Previous pediatric studies have shown that the rates of occlusions which fall in this range is low (10). All patients in this study had the position of the catheter confirmed via chest radiography, and the inflation volume determined using a previously validated inflation algorithm specific for pediatric patients (1), which is similar to published adult algorithms (11).

We randomly selected one test day for each patient with end-expiratory occlusion data and found that 56 patients “passed” with Pes/delta Paw between 0.8 to 1.2 during the OT, while 31 patients did not pass (35%). Patients who failed the OT had faster respiratory rate (33.5/min (26.3, 43.1), 27.0/min (22.0, 34.8), p<0.05), higher EL/Ers (0.89 (0.84, 0.92), 0.83 (0.78, 0.87), p<0.05), and lower pH (7.38(7.33, 7.41), 7.41(7.38, 7.43), p<0.05).

**Sensitivity Analysis 2: Pmusc and** ∆Pes**.**

When measuring ∆Pes, it does not segment lung and chest wall components, and specifically does not account for the thoracic recoil pressure. For this reason, respiratory muscle pressure (Pmusc) is thought to be the most accurate measure of patient effort, because it also factors in the inspiratory effort required against thoracic recoil by using C_CW,_ which is measured during an end-inspiratory hold. The limitation with Pmusc is that it requires an estimate of C_CW_, which mandates that the patient becomes passive during the end-inspiratory occlusion test. This has the potential to bias against patients with the highest respiratory effort or drive, who may not become passive with the inspiratory hold.

One test day per patient was randomly selected and we only selected test days with end-inspiratory holds that correctly measured chest compliance (no inspiratory or expiratory effort during inspiratory hold). Pmusc could not be measured on 25 patients, leaving 72 patients (72 random test days) for analysis. The correlation coefficient between Pmusc and ΔPes was 0.974 (p<0.001). Patients who could not have Pmusc calculated had higher PEEP 9.8 cmH_2_O (7.1-12.3), compared to those in whom Pmusc could be calculated 7.4 cmH_2_O (5.5-9.0)), p<0.05).

**Sensitivity Analysis 3: Effort on PC versus PS in SIMV**

SIMV is a mixed mode, with some breaths being time cycled and others being flow cycled. This can result in different breath durations from breath to breath, in addition to different levels of inspiratory pressure augmentation which is provided to the patient, based on how the ventilator is set. Hence, there remains some question about whether patient effort changes substantially from breath to breath during SIMV, based on whether the breath was assisted and time cycled (i.e. PC) versus supported and flow cycled (i.e. PS).

For this analysis, we calculated median ΔPes using up to 6 breaths, with a maximum of 3 breaths extracted for each PC and PS when patients were on SIMV mode. We found that there was minimal difference in patient effort (ΔPes) between PC and PS breaths in patients in SIMV mode are almost the same level (Figure E5).

References

1. Justin C Hotz, Cary T Sodetani, Jeffrey Van Steenbergen, Robinder G Khemani, Deakers TW, Newth CJ. Measurements Obtained From Esophageal Balloon Catheters Are Affected by the Esophageal Balloon Filling Volume in Children With ARDS. Respir Care 2018;63:177–186.

2. Al-Rawas N, Banner MJ, Euliano NR, Tams CG, Brown J, Martin AD, et al. Expiratory time constant for determinations of plateau pressure, respiratory system compliance, and total resistance. Crit Care 2013;17:R23.

3. Goligher EC, Dres M, Patel BK, Sahetya SK, Beitler JR, Telias I, et al. Lung- And diaphragm-protective ventilation. Am J Respir Crit Care Med 2020;202:950–961.

4. Kyogoku M, Shimatani T, Hotz JC, Newth CJL, Bellani G, Takeuchi M, et al. Direction and Magnitude of Change in Plateau From Peak Pressure During Inspiratory Holds Can Identify the Degree of Spontaneous Effort and Elastic Workload in Ventilated Patients. Crit Care Med 2020;49:517–526.

5. Chong WH, Saha BK, Medarov BI. Comparing Central Venous Blood Gas to Arterial Blood Gas and Determining Its Utility in Critically Ill Patients: Narrative Review. Anesth Analg 2021;133:374–378.

6. Moore DJ. The Assessment of Nutritional Status In Children. Nutrition Research 1985;5:797–799.

7. Mauri T, Yoshida T, Bellani G, Goligher EC, Carteaux G, Rittayamai N, et al. Esophageal and transpulmonary pressure in the clinical setting: meaning, usefulness and perspectives. Intensive Care Med 2016;42:1360–1373.

8. Baydur A, Behrakis PK, Zin WA, Jaeger M, Milic-Emili J. A simple method for assessing the validity of the esophageal balloon technique. American Review of Respiratory Disease 1982;126:788–791.

9. Milic-Emili J, Mead J, Turner JM, Glauser EM. Improved Technique for Estimating Pleural Pressure From Esophageal Balloons. J Appl Physiol 1964;19:207–211.

10. Okuda N, Kyogoku M, Inata Y, Isaka K, Moon K, Hatachi T, et al. Estimation of change in pleural pressure in assisted and unassisted spontaneous breathing pediatric patients using fluctuation of central venous pressure: A preliminary study. PLoS One 2021;16:1–11.

11. Mojoli F, Iotti GA, Torriglia F, Pozzi M, Volta CA, Bianzina S, et al. In vivo calibration of esophageal pressure in the mechanically ventilated patient makes measurements reliable. Crit Care 2016;20:1–9.

Table E1 Daily clinical parameters stratified by ventilator mode

|  | **ALL**  **(*N* =340)** | | | **< 1 year**  **(*N* = 55)** | | | **1 year to < 9 years**  **(*N* = 185)** | | | **>= 9 years to <= 18 years**  **(*N* = 100)** | | |
| --- | --- | --- | --- | --- | --- | --- | --- | --- | --- | --- | --- | --- |
|  | SIMV  n = 200 | PSV  n = 140 | p | SIMV  n = 35 | PSV  n = 20 | p | SIMV  n = 106 | PSV  n = 79 | p | SIMV  n = 59 | PSV  n = 41 | p |
| Peak Pressure (cmH_2_O) | 21.8  (18.1, 25.7) | 16.0  (13.5, 18.4) | < 0.001 | 21.1  (17.9, 24.1) | 14.3  (12.5, 16.3) | < 0.001 | 22.0  (17.9, 25.9) | 16.2  (13.8, 18.3) | < 0.001 | 21.5  (19.0, 26.9) | 16.1  (13.4, 18.4) | < 0.001 |
| Ppeak - PEEP (cmH_2_O) | 12.4  (10.1, 14.6) | 8.8  (5.9, 11.0) | < 0.001 | 12.5  (11.7, 16.2) | 7.0  (5.3, 10.0) | < 0.001 | 12.6  (10.0,14.5) | 9.8  (6.5, 11.2) | < 0.001 | 12.1  (9.8, 14.0) | 8.3  (5.3, 10.3) | < 0.001 |
| peak flow (L/s) | 0.29  (0.19, 0.49)  n = 197 | 0.27  (0.19, 0.35)  n = 134 | 0.419 | 0.16  (0.13, 0.17)  n = 33 | 0.14  (0.12, 0.16)  n = 20 | 0.117 | 0.26  (0.19, 0.34) n = 105 | 0.24  (0.20, 0.30) n = 76 | 0.773 | 0.63  (0.49, 0.79) n = 59 | 0.48 (0.31, 0.55) n = 38 | 0.304 |
| ∆Pes (cmH_2_O) | 7.2  (4.5, 11.9) | 9.7  (7.2, 13.3) | < 0.001 | 6.6  (3.6, 11.0) | 7.7 (5.3, 10.3) | 0.791 | 7.5 (4.7, 12.8) | 10.7 (7.9, 14.5) | < 0.001 | 6.8  (4.3, 11.9) | 8.7 (5.3, 12.6) | 0.006 |
| Pocc (cmH_2_O) | 12.7  (8.0, 17.0) n = 177 | 13.6 (9.7, 19.0) n = 126 | < 0.001 | 9.6 (7.6, 13.3) n = 32 | 12.1 (10.5, 15.5) n = 18 | 0.065 | 13.6  (7.5, 17.9) n = 95 | 14.2 (10.5, 20.5) n = 73 | < 0.001 | 13.3  (9.3, 17.9) n = 50 | 11.5 (6.8, 18.7) n = 35 | 0.125 |
| P0.1 (cmH_2_O) | 0.8  (0.5, 1.6) n = 177 | 0.8 (0.5, 1.5) n = 126 | 0.058 | 0.9  (0.6, 1.5) n = 32 | 0.7 (0.5, 1.1) n = 18 | 0.636 | 0.8  (0.5, 1.2) n = 95 | 1.0 (0.5, 2.0) n = 73 | 0.007 | 0.9  (0.5, 1.8) n = 50 | 0.8 (0.5, 1.1) n = 35 | 0.726 |
| PMI (cmH_2_O) | 0.6  (-0.9, 3.1) n = 159 | 2.9b (0.5, 5.5) n = 119 | < 0.001 | 0.1  (-1.1, 2.1) n = 26 | 3.2 (1.9, 6.4) n = 17 | 0.011 | 0.6  (-1.2, 3.3) n = 87 | 2.7 (0.7, 4.9) n = 67 | < 0.001 | 1.3  (-0.8, 3.1) n = 46 | 4.4 (0.1, 6.4) n = 35 | < 0.001 |

Table E2 Correlation coefficient between ∆Pes and Pocc/P0.1/PMI on PSV mode

|  | **ALL**  **(*N* = 140)** | **< 1 year**  **(*N* = 20)** | **1 year to < 9 years**  **(*N* = 79)** | **>= 9 years to <= 18 years**  **(*N* = 41)** |
| --- | --- | --- | --- | --- |
| Pocc (cmH_2_O) | r = 0.59 (95% CI: 0.46, 0.67) n = 126 | r = 0.89  (95% CI: 0.59, 0.97) n = 18 | r = 0.56 (95% CI: 0.38, 0.70) n = 73 | r = 0.62 (95% CI: 0.39, 0.76) n = 35 |
| P0.1 (cmH_2_O) | r = 0.48 (95% CI: 0.18, 0.68) n = 126 | r = 0.78 (95% CI: 0.12, 0.98) n = 18 | r = 0.49 (95% CI: 0.24, 0.76) n = 73 | r = 0.17 (95% CI: -0.20, 0.44) n = 35 |
| PMI (cmH_2_O) | r = 0.45 (95% CI: 0.24, 0.63) n = 119 | r = -0.06 (95% CI: -0.50, 0.91) n = 17 | r = 0.59 (95% CI: 0.25, 0.74) n = 67 | r = 0.41 (95% CI: -0.19, 0.75) n = 35 |

Table E3 Correlation coefficient between ∆Pes and Pocc/P0.1/PMI using patient days with delta Pes/delta Paw 0.8 to 1.2 in end-expiratory occlusion

|  | **All ventilator mode** | | | | **PSV mode** |
| --- | --- | --- | --- | --- | --- |
|  | All  (N=144, 64 patients) | < 1 year  (N= 21, 11 patients) | 1 year to <9 years  (N= 74, 30 patients) | >= 9 years to <= 18 years  (N = 49, 23 patients) | All  (N = 61, 33 patients) |
| Pocc | r = 0.72  (95% CI: 0.60, 0.82)  (n= 144) | r = 0.84  (95% CI: 0.38, 0.96 )  (n= 21) | r = 0.68  (95% CI: 0.48, 0.82)  (n= 74) | r = 0.76  (95% CI: 0.52, 0.89)  (n= 49) | r = 0.53  (95% CI: 0.18, 0.66)  (n= 61) |
| P0.1 | r = 0.35  (95% CI: 0.14, 0.53)  (n= 144) | r = 0.58  (95% CI -0.13, 0.90)  (n= 21) | r = 0.31  (95% CI: 0.01, 0.56)  (n= 74) | r = 0.42  (95% CI: 0.02, 0.70 )  (n= 38) | r = 0.16  (95% CI: -0.30, 0.50)  (n= 61) |
| PMI | r = 0.42  (95% CI: 0.19, 0.61)  (n= 120) | r = 0.6  (95% CI: -0.28, 0.93)  (n= 17) | r = 0.35  (95% CI: 0.02, 0.62)  (n= 65) | r = 0.51  (95% CI: 0.06, 0.79)  (n= 38) | r = 0.32  (95% CI: -0.04, 0.63)  (n= 61) |
